# N-acetylcysteine in COPD: why, how, and when?

**DOI:** 10.1186/s40248-016-0039-2

**Published:** 2016-02-03

**Authors:** Claudio M. Sanguinetti

**Affiliations:** Respiratory Medicine, Quisisana Clinical Center, Rome, Italy

**Keywords:** COPD, Oxidative stress, Exacerbations, Antioxidants, High dose oral N-acetylcysteine

## Abstract

Oxidants have long been recognized to have an important role in the pathogenesis of COPD, and in this cigarette smoke has a strong responsibility, because it generates a conspicuous amount of oxidant radicals able to modify the structure of the respiratory tract and to enhance several mechanisms that sustain lung inflammation in COPD. In fact, oxidative stress is highly increased in COPD and natural antioxidant capacities, mainly afforded by reduced glutathione, are often overcome. Thus an exogenous supplementation of antioxidant compounds is mandatory to at least partially counteract the oxidative stress. For this purpose N-acetylcysteine has great potentialities due to its capacity of directly contrasting oxidants with its free thiols, and to the possibility it has of acting as donor of cysteine precursors aimed at glutathione restoration. Many studies *in vitro* and *in vivo* have already demonstrated the antioxidant capacity of NAC. Many clinical studies have long been performed to explore the efficacy of NAC in COPD with altern results, especially when the drug was used at very low dosage and/or for a short period of time. More recently, several trials have been conducted to verify the appropriateness of using high-dose NAC in COPD, above all to decrease the exacerbations rate. The results have been encouraging, even if some of the data come from the most widely sized trials that have been conducted in Chinese populations. Although other evidence should be necessary to confirm the results in other populations of patients, high-dose oral NAC nevertheless offers interesting perspectives as add-on therapy for COPD patients.

## Background

Chronic Obstructive Pulmonary Disease (COPD), a common, preventable and treatable disease, is characterized by a chronic airflow obstruction that is usually progressive and scarcely reversible,caused by a variable association of chronic bronchitis, small airways damage and pulmonary emphysema, consequent to inhalation of noxious particles and gases, especially tobacco smoke, inducing a chronic airway inflammation, and frequently associated with several comorbidities [1, 2].

The characteristic pathological changes in the lungs of COPD patients are sustained by an inflammation where a pivotal role is exerted by excessive oxidant stress and protease imbalance [3, 4].

A wide variety of oxidants, free radicals and others agents are implicated in the pathogenesis of COPD, thus the administration of antioxidants seems a rational adjunct to the treatment of the disease and in this respect the oral administration of N-acetylcysteine (NAC) proved effective in decreasing oxidative biomarkers in peripheral blood and exhaled breath condensate, and in reducing the rate of COPD exacerbations [5–9].

## Role of oxidative stress in COPD pathogenesis

COPD is a chronic disease, presenting with different phenotypes and frequent exacerbation episodes, based on a pulmonary and extrapulmonary inflammation with increase also in reactive oxygen species (ROS). Oxidants have long been recognized to have an important role in the pathogenesis of COPD [3, 4, 10–12], because, besides a direct lung damage, they also enable the activation of transcription factors like nuclear factor NF-*k*B with consequent production of inflammatory proteins and damage of antiproteases [13].

Oxidant compounds are highly reactive oxygen radicals, originated from the reduction of molecular oxygen in the course of physiological and pathological processes, able to induce a chemical reaction (redox cycle) where the oxidant takes up electrons from another compound and then transfers them to other molecules so inducing structural and functional alterations. In biological processes the true radicals mainly involved are superoxide, hydroxyl, hydroperoxyl, nitrogen dioxide, while non radicals compounds like singlet oxygen, ozone, and hydrogen peroxide can also be important by generating other radicals [3].

Oxygen free radicals, having various and different cellular penetration and activity [14], can substantially recognize exogenous and endogenous origin. Air pollutants, both gaseous and particulate matter, are a major exogenous source of oxidants through the formation of ROS that, overwhelming antioxidant defences, can react with DNA, lipids and proteins, so inducing short-term and long-term effects by activating signaling pathways, enhancing inflammatory and degenerative processes, and impairing the protective mechanisms [15]. Oxidants may also derive from the redox cycle of drugs or toxins, and in case of exposition to hyperoxia. Agents like bleomycin, alloxan, doxorubicin, paracetamol and others are able to generate superoxide anions and hydroxyl radicals [3].

Among the exogenous sources of oxidants, cigarette smoke has a prominent position because it contains a very high amount of radicals per puff [16] and it can also activate inflammatory cells to deliver ROS. In fact, the oxidant load in the respiratory tract is further augmented due to the increased amount of inflammatory cells at this site, and a close correlation has been demonstrated by cigarette smoking and neutrophils production, increased circulation, and sequestration in lung capillaries [17]. After sequestration, neutrophils are chemotactically attracted to the airways and lung parenchyma where they can deliver powerful oxidants and proteases [18]. Macrophages are also activated by cigarette smoke to release inflammatory mediators and ROS [19]. Thus, cigarette smoking, a very frequent habit in COPD patients, has to be considered the main contributing factor to oxidative stress in these patients. Other than exerting a direct injury to the lungs, oxidant radicals can also stimulate or exacerbate other important mechanisms like inflammation, protease/antiprotease imbalance and apoptosis [3, 20–23].

Physiological processes in the body are an endogenous source of free radicals with the complete reduction of an oxygen molecule to water. It is critical for cellular respiration, because the mitochondrial electron transport chain originates hydrogen peroxide. The toxic compounds so generated are usually completely balanced by the antioxidant defenses. Apart from these physiological processes, a strong endogenous source of ROS in the lungs is represented by inflammatory cells, like neutrophils and eosinophils, and by macrophages, epithelial and endothelial cells, all of which can also be activated by the inhalation of exogenous pollutants, like particulate matter, sulphur dioxide and nitrous dioxide, to produce ROS, mainly superoxide anions and hydrogen peroxide [24, 25]. Some transcriptional factors implicated in inflammatory processes, like NF-*k*B and activator protein-1 (AP-1) are activated by oxidative stimuli and mediate the expression of cytokine and other biological agents [26, 27].

The term “oxidative stress”, clearly demonstrated in smokers and in COPD patients [20, 28], specifically means an imbalance between the burden of oxidants in the lung and the amount of antioxidants in favor of the former, mainly caused by cigarette smoking and potentially able to endanger the lung structure and function. Following the exposure to toxins and cigarette smoke, alveolar epithelium releases chemotactic agents for inflammatory cells recruitment and consequent lung damage [29]. The lung matrix (elastin, collagen) and the alveolar epithelial surface directly exposed to inhaled pollutants can be damaged especially when the protective activity of intra- and extracellular glutathione is overwhelmed by oxidant burden [30].

The attack of oxidants to fatty acids of cellular membranes triggers the process of lipid peroxidation with generation of hydroperoxides and other unstable compounds that can amplify the reaction. Such compounds are increased in smokers and in COPD patients [7, 10, 31, 32] and testify close interrelationships between markers of inflammation and oxidative mechanisms in the pathogenesis of COPD [33–35].

Furthermore, a positive correlation has been demonstrated between the value of forced expiratory volume in one second (FEV_1_) and antioxidant level in plasma [36, 37], whereas antioxidant depletion was more frequent in severe COPD patients with frequent exacerbations [38].

## Endogenous and exogenous antioxidant defenses

Antioxidant protection in the tissues and also in the respiratory tract is mainly provided by the glutathione (GSH), a redox-cycler thiol present in epithelial lining fluid, that, by transforming from the reduced to oxidized form, exerts a powerful antioxidant effect and then is converted again to the reduced form by the enzyme glutathione reductase. The antioxidant action of glutathione associates also with some enzymatic activities, like aldehyde dehydrogenase and superoxide dismutase, able to counteract the oxidant and inflammatory reactions of cigarette smoke [37, 39]. Among the endogenous defenses a distinct role has also the transcription factor nuclear erythroid-related factor 2 (Nrf2) that, in presence of excessive oxidant burden, translocates from the cytoplasm to the nucleus enhancing the production of antioxidant genes [40–42].

All these endogenous antioxidant mechanisms can be overcome in COPD patients, mainly in strong smokers or ex-smokers, by an increased oxidative stress [3, 10, 28]. As a matter of fact, GSH content in alveolar cells was shown to be reduced following acute exposure to cigarette smoke in animal experiments [43] and its levels were also reduced in COPD patients due to a downregulation of glutathione biosynthetic enzymes [42] whose content indirectly correlates with the severity of the respiratory disease [41]. The oxidative stress can also reduce the activity of the enzyme histone deacetylase-2, thus enhancing the transcription of proinflammatory genes and inducing a corticosteroid resistance [44, 45]. COPD exacerbations further aggravate this situation as demonstrated by the decreased level of GSH in bronchoalveolar lavage fluid during acute episodes compared to stable state [46, 47].

As to the exogenous antioxidant supply, the results of many investigations are not always concordant. Epidemiological studies carried out many years ago in general population showed a small but significant increase in respiratory function correlated with a dietary intake of vitamin C and less likely of vitamin E [48]. These results were confirmed some years later in a wide Dutch study, where the intake of vitamin C and beta-carotene was associated with better respiratory function values [49]. However, the supplementation of vitamin antioxidants did not result in improved symptoms or respiratory function in almost thirty thousand COPD patients [50], whereas in another study a protective role for vitamin C against the risk of obstructive airways disease has been demonstrated, probably due to the counteracting action of this vitamin on the effects of cigarette smoking [51]. Even a high intake of catechins, a subclass of antioxidant flavonoids, and of solid fruits was proven to exert a beneficial effect against COPD [52]. Resveratrol is a polyphenol synthesized by some vegetal species that displays various biological activities among which anti-inflammatory and antioxidant properties [53–55].

However, the reduced glutathione system is the mainstay of the defenses against oxidative stress and in case of excessive consumption of GSH its restoration cannot be provided only by dietary supply, and exogenous supplementation with thiol groups donors or compounds enhancing the GSH synthesis may be necessary. In this respect N-acetylcysteine (NAC), a substance whose efficacy has been in the past almost exclusively attributed to its mucolytic properties [56, 57], has eventually been demonstrated able to counteract oxidant burden both directly by its thiolic free groups acting in the extracellular environment [58], and indirectly by providing intracellularly cysteine for GSH synthesis [59].

## The basis for N-acetylcysteine as an antioxidant and anti-inflammatory agent

The protection afforded by NAC against oxidants has been recognized many decades ago, even if its bioavailability when administered orally was not fully described and the main useful effect of the drug was more recently recognized as precursor of GSH [60]. N-acetylcysteine when administered orally is deacetylated to cysteine, with consequent increase in the concentration of reduced glutathione in plasma and airways, as demonstrated by the increase of GSH in bonchoalveolar lavage fluid after administration of NAC, and by the active uptake of glutathione from plasma by the lungs, that are the main place of GSH synthesis together with the liver [61, 62]. A study performed some years later was able to confirm the increase of GSH in plasma of COPD patients, while it was not increased in bronchoalveolar lavage fluid, after high daily doses of NAC when administered for a short period of time, in this case for five days only [63].

However, a protection of the alveolar epithelium from oxygen toxicity by NAC was shown long time ago both *in vitro* and *in vivo* in animal experiments [64, 65]. The free thiol group of NAC can interact with electrophilic residual of reactive oxygen species with subsequent formation of intermediate compounds like NAC disulphide [59, 60, 66, 67]. Direct administration of L-cysteine is impaired by its low intestinal absorption and rapid metabolism in the liver, while NAC, rapidly absorbed after oral administration, allows to overcome these disadvantages [67–69] and to restore the intracellular GSH decreased by the oxidative stress and inflammatory processes [70–72]. Exogenous NAC was able to restore intracellular content of thiols, whereas GSH administration was not [69, 73]. Only a limited plasma increase in GSH was recorded in healthy subjects given 600 mg NAC [74], demonstrating a clear use of NAC *per os*.

Some studies have demonstrated a dose-dependent effect of NAC in COPD patients, because the usual dose of 600 mg once daily was not able to increase GSH, while it occurred when NAC was administered three times a day [63]. Owing to its antioxidant properties, NAC is able to maintain the redox-dependent cell-signaling and transcription, in particular the Nuclear Factor–kB (NF-kB), p38 MAPK and others, critical for proinflammatory genes regulation [75, 76]. This favourable effect of NAC may be induced by low NAC doses if the administration period is sufficiently prolonged, but it can be more rapidly achieved with higher NAC daily doses chronically given, that likely exert a more marked control on the activation of inflammatory factor like NF-kb [69], demonstrating a useful use of high dose of NAC *per os*.

More recently, a new mechanism of NAC action has been demonstrated in animals exposed to cigarette smoke, concerning the positive effect of NAC on nuclear erythroid 2–related factor–2 (Nrf2) transcription factor that has a crucial role as regulator of cellular redox status [77] also in COPD patients where it has been shown to be decreased [41, 78].

## Clinical studies of NAC administration in COPD patients

There are in the literature many studies that found beneficial effect of NAC in humans in terms of preserving oxidant/antioxidant homeostasis through the increase in GSH, the decrease in the content and activation of inflammatory cells in sputum and in BAL [33, 61, 79–81] but also, many trials that investigated the effect of oral administration of NAC on the ROS production as evidenced in exhaled breath condensate (EBC).

About twenty years ago Dekhuijzen [10] first revealed an increased content of oxidants (hydrogen peroxide,H_2_O_2_) in EBC of clinically stable COPD patients compared to healthy subjects, and the H_2_O_2_ level was even significantly higher in exacerbated COPD patients than in the stable ones. The same results were obtained by Novak et al. [82]. Subsequently, De Benedetto et al. [83], using a new technique allowing to minimize the inaccuracy deriving from the instability of hydrogen peroxide, assessed the level of this compound in EBC of normal subjects and of stable patients with mild to moderate COPD, confirming that mean H_2_O_2_ level in exhaled air of COPD patients (0.50 ± 0.11SD μM) was significantly (*p* = 0.0001) increased compared to that in healthy subjects (0.12 ± 0.09 μM). Thus, even in a stable clinical condition, COPD is sustained by chronic inflammatory processes also involving oxidant production, and this production is even more increased during exacerbation episodes [10].

Several trials in the past investigated the usefulness of oral administration of NAC, generally 600 mg/day in chronic bronchitis and COPD patients above all to prevent exacerbations [8, 84–86], obtaining positive results [8], even if some studies only observed a positive clinical trend without statistical correspondence [86].

At the beginning of the present century Stey et al. [9] performed a systematic review of 11 studies published until 1994, where NAC had been compared to placebo in over 2 thousand COPD patients, and the results were in favor of NAC, whose administration for 12 to 24 months decreased the rate of exacerbations and the symptoms score, irrespective of duration of therapy or of cumulative dose administered. The same year, another meta-analysis [87] conducted on 8 studies qualified for inclusion published until 1995, with NAC administered orally at a maximal dose of 600 mg/day in six studies, at 1200 mg/day in one study, and at 1800 mg/day in another one, confirmed the beneficial effect of N-acetylcysteine on exacerbation rate. A further meta-analysis performed the following year, focusing on the effect of mucolytics in COPD and chronic bronchitis, including 12 studies with NAC out of a total of 23, concluded that treatment with these drugs is associated with a reduction of acute exacerbations and days of illnesses in these patients [88].

In a wide, multicentric trial [89] more than 5 hundred COPD patients were randomized to receive NAC 600 mg/day or placebo for three years. The yearly rate of decline in FEV_1_ was not decreased in patients given N-acetylcysteine compared to those on placebo, as well as the exacerbation rate, probably because a single dose of 600 mg is not sufficient to cause modifications of these outcomes, even if the frequency of exacerbations was significantly lower in COPD patients treated with NAC and without inhaled corticosteroids. A similar finding was evidenced by Sutherland et al. [90] in a meta-analysis of 8 trials from which derived that NAC reduces the risk of exacerbations in patients with COPD and this effect can be attenuated by inhaled steroids but not by smoking. This probably demonstrates that, besides the adequate dosage and duration of NAC administration, also a careful phenotyping of COPD patients is critical for reaching the greatest benefit in terms of symptoms control and exacerbation prevention. This is likely also the reason why some studies performed in primary care patients affected with COPD or chronic bronchitis did not evidence any advantage from the administration of NAC even for a long period but at a dose of 600 mg daily [91].

**Fig. 1:**
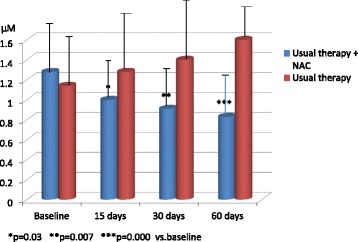
Mean values of H_2_O_2_ in expired breath condensate (EBC) of COPD patients, treated with usual therapy plus NAC or with usual therapy only, before the study and at 15, 30, and 60 days after the start of the study. Mod from [7]

On the contrary, Kasielsky and Novak [92] had previously demonstrated that 600 mg/day of NAC for 12 months are able to significantly decrease the hydrogen peroxide level in EBC of COPD patients compared to placebo, but only after the sixth month of NAC administration. On the same line, three years later De Benedetto et al. [7] investigated the effect of NAC 1200 mg given for two months together with usual therapy to stable patients affected with moderate COPD in comparison to another group of patients given the usual therapy only, and in both groups inhaled corticosteroids (iCS) were suspended at the beginning of the trial. The assay of H_2_O_2_ level in EBC was performed before the beginning of NAC administration and at 15, 30 and 60 days after the start of NAC therapy. As shown in Fig. 1, in patients treated with the usual therapy plus NAC significantly lower concentrations of H_2_O_2_ were measured in EBC at 15,30 and 60 days compared to the values recorded before the beginning of the treatment. The EBC H_2_O_2_ values were instead increased at the same time points compared to the basal value in COPD patients treated with usual therapy only, likely due to a lack of control of airways chronic inflammation by iCS and increased ROS production.

This study [7] also further points out that, in order to efficiently counteract oxidative stress in COPD patients, and decrease exacerbations rate and hospitalization [93], higher NAC doses are needed than those used in past investigations, generally limited to 600 mg once a day.

## High-dose NAC supplementation in COPD patients

Based on the fact that lower NAC doses not always yielded consistent and homogeneous results, and in addition a dose-dependence of NAC was demonstrated [69], several studies in last years focused on the effectiveness of high-dose NAC (1200 mg/day) as add on therapy to the usual one in COPD, especially with the aim to decrease symptoms, prevent exacerbations, and possibly improve their treatment.

For this purpose Zuin et al. [94] investigated the efficacy and tolerability of high-dose NAC during the course of an acute COPD exacerbation, in a double-blind, double-dummy, placebo-controlled study including 123 patients. Patients were randomly assigned to: a group given NAC 1200 mg/day for 10 days in addition to usual therapy (inhaled corticosteroids, theophylline, anticholinergics and beta-2 adrenoceptor agonists); or a group given NAC 600 mg/day for 10 days in addition to usual therapy; or a group given placebo plus usual therapy for 10 days. The anti-inflammatory effect was assessed by the level of serum C-reactive protein (CRP) and interleukin-8 (IL-8), while FEV_1_ and respiratory symptoms score were also recorded. Patients were examined at screening visit and at 5 and 10 days of treatment. In patients with abnormal values of CRP, the normalization of this index occurred in 90 % of patients given NAC 1200 mg/day, in 52 % of those given 600 mg/day, and in 19 % of placebo patients. The differences of both NAC treatments were statistically significant vs. placebo, but NAC 1200 was also significantly different from NAC 600 (*p* = 0.002). Furthermore, serum IL-8 was decreased at 10 days only after administration of NAC 1200 mg/day, whereas FEV_1_ value showed small but significant increase after both doses of NAC, so as the symptoms improvement. NAC was well tolerated also at the higher dose. The Authors concluded that a daily dose of NAC 1200 mg improves the clinical outcomes in patients with exacerbations of COPD.

An interesting, recent investigation [95] used new imaging tools to verify the effect of high-dose NAC in COPD patients, finding a correlation between the image-based improvement in respiratory function, mainly a better functionality of small airways with consequent decrease in lung hyperinflation, and effects of NAC supplementation as witnessed by the glutathione increase.

The beneficial effects of high-dose NAC (1200 mg/day) to prevent COPD exacerbations was investigated in 2013 in a systematic review and meta-analysis, based on 11 studies, performed by Shen et al. [96]. The high-dose of NAC proved effective for the purpose of preventing exacerbations, whereas the lower dose demonstrated some efficacy only in studies with high methodological quality. In addition, both doses did not show any influence on respiratory function.

A Chinese trial (HIACE) published the same year by Tse et al. [97] confirmed the efficacy of high-dose NAC against exacerbations in COPD patients, but revealed also a beneficial effect on the small airways function, as revealed by De Backer [95] with original imaging techniques. The HIACE trial (The Effect of High Dose N-acetylcysteine on Air Trapping and Airway Resistance of Chronic Obstructive Pulmonary Disease—a Double-blinded, Randomized, Placebo-controlled Trial) was conducted in Hong-Kong on 120 patients randomly assigned to take NAC 600 mg b.i.d. or placebo plus usual therapy for one year. At the end of trial, the frequency of COPD exacerbations with NAC was significantly lower than with placebo, and the value of mean forced expiratory flow (FEF_25-75_) was slightly but significantly increased in NAC group compared to the placebo one, so as the reactance and resistance obtained with forced oscillation technique (FOT). Mean expiratory flow on maximal flow-volume curve, long recognized as a functional marker of small airways [98], has a great variability and presents substantial interpretative problems [99–104], whereas low-frequency resistance and reactance measured with the FOT would be more reliable to functionally explore the small airways [105–107]. The improvement of respiratory function with the long-term administration of high-dose NAC (significant increase in FOT reactance at 6 Hz and trend to decrease of resistance at 6 Hz compared to placebo) is attributed by Authors [97] to an effective antioxidant and anti-inflammatory effect of the drug on small airways, and consequently also on lung hyperinflation, as already found [95, 108], due to the adequacy of dose and length of therapy, differently from previous studies were the dosage used was too low and/or the duration of therapy was too short for the drug to be effective [63, 66, 92]. The improvement of respiratory function would be another possible beneficial effect of high-dose NAC that, while needing further confirmation in larger studies, opens new and interesting perspectives for the use of this drug in COPD treatment. More established seems the effect of high-dose NAC as protective from COPD exacerbations, whose frequency in this study was 0.96/year with NAC vs. 1.71/year with placebo (*p* = 0.019), and this is possibly due, other than to the antioxidant and antinflammatory effects, also to the capacity of NAC to interfere with the bacterial functionality as demonstrated more in general by mucolytic agents [97, 109]. Also a non-significant trend towards a decreased rate of re-hospitalization with NAC administration emerged from HIACE trial, as already demonstrated in another study [93] and this is important not only to prevent further progression of the disease, but also to contain the major source of costs of COPD. Similarly important for its practical implications appears also the demonstrated capacity of NAC to increase the patient’s tolerability to exercise, that is frequently reduced in COPD patients [110, 111]. No safety problems were recorded also with this high dosage.

**Fig. 2:**
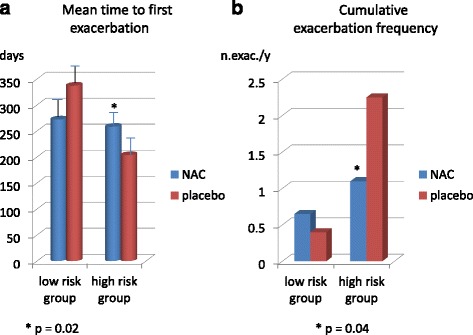
**a** Mean time to first exacerbation; **b** mean cumulative exacerbation frequency in Chinese COPD patients treated with high-dose NAC or placebo in adjunct to usual therapy for one year and divided according to basal high or low exacerbation risk. Mod. from [111]

**Fig. 3:**
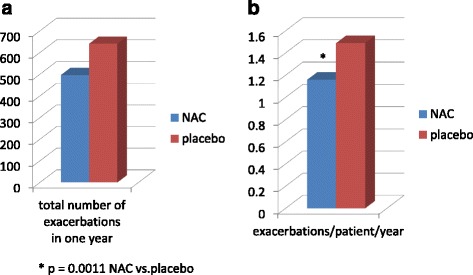
**a** Total number of COPD exacerbations occurred in one year in NAC group and in placebo group; **b** number of exacerbations/patient/year in the two groups of patients. Mod from [119]

The following year the same group of investigators made a *post-hoc *analysis [112] of their series focused on the efficacy of NAC as protection from COPD exacerbations according to the baseline patient’s exacerbation risk as determined by GOLD criteria. Thus, patients were divided in two groups, low and high risk of exacerbations, and in the high risk group NAC, compared to placebo, was able to significantly increase the time to first exacerbation, decrease the exacerbation frequency and augment the probability of being exacerbation-free at the end of one-year treatment (51.3 % of patients on NAC vs. 24.4 % of patients on placebo; *p* = 0.013) (Fig. 2). No significant benefit was achieved with NAC administration in low risk group. The history of frequent exacerbations and the severity of COPD are important to define a group of patients present in all severity stages of GOLD classification [113] who present the greatest functional decline, the major number of comorbidities and are exposed to a poorer outcome and death [114]. Thus, the possibility of beneficially affecting the exacerbation frequency with high-dose NAC given for a long period of time, in addition to other drugs that proven useful for this purpose [115–118], appears an important clue for the adequate treatment of COPD patients affected with more severe disease, as already demonstrated in other studies with high-dose mucolytics [119].(Fig. 3)

Indeed, in the same study, performed on Chinese patients [112], authors themselves raise some doubts about the generalization of their results to non-Chinese white populations, but underline that NAC was effective also in high-risk patients treated with inhaled corticosteroids, differently from previous findings [89].

This topic has been further investigated in another wider Chinese study (the Placebo-controlled study on efficAcy and safety of N-acetylcysTeine High dose in Exacerbations of chronic Obstructive pulmoNary disease [PANTHEON] study) [120], where high-dose NAC or placebo were administered for one year, in addition to existing individual therapy, to patients with stable, moderate-to-severe COPD, and reporting at least 2 exacerbation episodes during the last two years, stratified by previous use of inhaled corticosteroids or not at the beginning of the study. The primary outcome variable was the rate of COPD exacerbations in one year, while the secondary variables were the time to first exacerbation, the amount of patients with severe exacerbations (requiring systemic corticosteroids and antibiotics), and the need for rescue bronchodilator therapy. One thousand and six patients were randomly allocated to NAC (504) or placebo (502), without between groups differences in baseline characteristics, and the duration of the study was the same for both groups (319 days). At the end of the study the number of exacerbations was higher in placebo group (641 episodes) than in NAC group (497 episodes), which corresponds to a mean of 1.49 exacerbations/patient/year with placebo vs. 1.19 exacerbations/patient/year with NAC (*p* = 0.0011), with a decrease in exacerbation risk of 22 % determined by NAC. N-acetylcysteine induced a significantly lower frequency of severe exacerbations and also decreased the duration of the exacerbation compared to placebo as already found in other studies [94], whereas previous ICS use did not seem to influence the treatment effect, as already found in another chinese trial [97, 112]. There was no difference in the time to first exacerbation between groups, but it resulted reduced in patients with moderate disease (GOLD II) treated with NAC, which also lengthened the time to second and third exacerbation compared to placebo in the whole study group. NAC was well tolerated by COPD patients also in this study.

The results of PANTHEON study are thus in accordance with those of previous trials investigating the prevention of exacerbations afforded by NAC [97, 119]. Authors of the PANTHEON trial also underline the greater effect of NAC in patients with GOLD II disease, thus suggesting that the antioxidant should be given at an earlier stage of disease, to contrast the COPD progression towards more severe and irreversible alterations, and for a long time in order to attain the greatest efficacy from treatment. This draws further attention to the necessity of knowing the type of COPD patients where NAC can have the greatest effect [121].

The PANTHEON study raised some criticism [122], firstly concerning the definition of exacerbation as used in this trial. In fact, the occurrence of an exacerbation was assessed based on daily symptoms recorded by patients, that is a quite easy but possibly deceptive method. Besides, it was also noted that the phenotypes of patients included in the study group could be different from those of non-Chinese populations. The authors of the PANTHEON trial rebutted that, even supposing an under-reporting by patients, because of randomization its proportion should be similar for both groups therefore without any influence on the observed differences. On the other hand, mild exacerbations, that are recognized to be the most conspicuous portion of total exacerbations [123], and in PANTHEON studies accounted for 32 % of total, are only diagnosed by an increase in habitual symptoms and generally do not require a more intensive therapy or hospitalization.

In a comment on high-dose N-acetylcysteine in COPD Cazzola and Matera [124] do not exclude the potential usefulness of NAC for the treatment of COPD patients, even if they rely more on the mucolytic properties of the drug than on its antioxidant effect. Furthermore, these authors once more point out the lack in PANTHEON trial of a phenotypization of patients that likely would have clarified the exact role of the antioxidant therapy in COPD, as already demonstrated in the *post-hoc* analysis of HIACE study where the efficacy against exacerbations occurred only in the high-risk COPD patients, that is a well defined group of patients where the disease has a more severe connotation and poorer outcome.

However, in recently published evidence-based guidelines on the prevention of acute exacerbations of COPD by American College of Chest Physicians and Canadian Thoracic Society, N-acetylcysteine therapy is also recommended (grade 2B) for patients with moderate to severe COPD and a history of two or more exacerbations in the previous 2 years [125]. This evidence is also present in the GOLD recommendations [2].

## Conclusions

The title of this review puts some questions about adding N-acetylcysteine to the treatments of COPD.

The first question is why N-acetylcysteine should be added to other drugs routinely used at present in patients with COPD. The antioxidant and anti-inflammatory properties of NAC are based on a consistent amount of proofs obtained *in vitro* and *in vivo* and new information is continuously yielded about the useful involvement of this drug into the most intimate mechanisms playing a decisive role in triggering and maintaining respiratory inflammation. The inflammatory nature of COPD has already been well demonstrated [126], so as that oxidative stress has a close inter-relationship with the inflammatory process [5, 11, 20, 21, 33]. Moreover, the ROS level in the lung is steadily increased in COPD patients [10, 83]. NAC has the potential to interfere with the processes that underline COPD pathogenesis and, as a matter of fact, the respiratory level of oxidants is decreased after its administration [7]. Thus, the antioxidant and mucolytics properties of NAC have the potential for being a useful adjunct in COPD therapy [57, 69]. Even because a recent investigation has demonstrated that NAC is also able to enhance the effectiveness of antimuscarinic bronchodilators generally used in COPD patients [127].

The second question, that is how to administer N-acetylcysteine, seems just an overcome issue, because the numerous trials that have investigated NAC demonstrated that oral route is efficient, simply to use, and well tolerated, without excluding however other potential modalities of administration. The daily dosage of the drug raised some perplexities in the past, but it is well established by now that to achieve some benefit in terms of prevention from COPD exacerbations or possibly of respiratory function and symptoms improvement, high-dose NAC (1200 mg/day on average) must be administered for a long period of time. All the most recent trials used this dosage for one year to evaluate the effect of NAC in adjunct to usual therapy in comparison to placebo.

Finally, the most critical point, still matter of discussion, is when and to which COPD patients NAC therapy should be addressed. The results of widely sized studies performed to assess the value of high-dose adjunct of NAC or other mucolytic agents to COPD therapy are encouraging, but they were obtained in Chinese patients [97, 119, 120] and the limits to apply these results are above underlined. However, these studies also evidenced important and seemingly conflicting results, because one revealed that high-dose NAC reaches its greatest efficacy in patients characterized by a high risk of being exacerbated, whereas the other one revealed more NAC efficacy in those patients who still are in a phase of moderate gravity of the disease. Likely, this discrepancy in results may depend on the composition of the enrolled patients in these studies, and they can clearly convey the need for a better phenotyping of COPD patients to investigate, as already come out for other respiratory drugs like bronchodilators and inhaled corticosteroids. Furthermore, these results could assign to NAC a broader role than previously recognized, that is that of a drug able to be used as add on therapy to decrease the exacerbation rate and symptoms score, represented by cough and phlegm, in COPD patients, also without the moderate to severe airways obstruction.

In spite of the above mentioned limits, the results of past and recent studies can offer very important perspectives for the use of oral NAC as a valid add on therapy in chronic bronchitis and COPD and other widely sized clinical trials in different populations are hoped for in order to establish the role of high-dose NAC in the therapy of COPD patients.

## References

[1] Bettoncelli G, Blasi F, Brusasco V, Centanni S, Corrado A, De Benedetto F (2014). The clinical and integrated management of COPD. An official document of AIMAR (Interdisciplinary Association for Research in Lung Disease), AIPO (Italian Association of Hospital Pulmonologists), SIMER (Italian Society of Respiratory Medicine), SIMG (Italian Society of General Medicine). Multidiscip Resp Med.

[2] Global Strategy for the Diagnosis, management and prevention of chronic obstructive pulmonary disease (updated 2015). www.goldcopd.org (accessed May 2015).

[3] Sanguinetti CM (1992). Oxidant/antioxidant imbalance: role in the pathogenesis of COPD. Respiration.

[4] Rahman I. Oxidative stress in pathogenesis of chronic obstructive pulmonary disease: cellular and molecular mechanisms. Cell Biochem Biophys. 2005;43:167–88. 10.1385/CBB:43:1:167 16043892

[5] Rahman I. Antioxidant therapies in COPD. Int J Chron Obstruct Pulmon Dis. 2006;1:15–29. 10.2147/copd.2006.1.1.15 PMC2706605 18046899

[6] Van Schooten FJ, Nia AB, De Flora S, D’Agostini F, Izzotti A, Camoirano A (2002). Effects of oral administration of N-acetyl-L-cysteine: a multi-biomarker study in smokers. Cancer Epidemiol Biomarkers Prev.

[7] De Benedetto F, Aceto A, Dragani B, Spacone A, Formisano S, Pela R (2005). Long-term oral N-acetylcysteine reduces exhaled hydrogen peroxide in stable COPD. Pulm Pharmacol Ther..

[8] Pela R, Calcagni AM, Subiaco S, Isidori P, Tubaldi A, Sanguinetti CM (1999). N-acethylcysteine reduces exacerbation rate in patients with moderate to severe COPD. Respiration.

[9] Stey C, Steurer J, Bachmann S, Medici TC, Tramer MR (2000). The effect of oral N-acetylcysteine in chronic bronchitis: a quantitative systematic review. Eur Respir J.

[10] Dekhuijzen PNR, Aben KK, Dekker I, Aarts LP, Wielders PL, van Herwaarden CL (1996). Increased exhalation of hydrogen peroxide in patients with stable and unstable chronic obstructive pulmonary disease. Am J Respir Crit Care Med.

[11] Repine JE, Bast A, Lankhorst I (1997). Oxidative stress in chronic obstructive pulmonary disease. Am J Respir Crit Care Med.

[12] Barnes PJ (2000). Chronic Obstructive Pulmonary Disease. N Engl J Med.

[13] Barnes PJ, Karin M (1997). Nuclear factor-*k*B — a pivotal transcription factor in chronic inflammatory diseases. N Engl J Med.

[14] Pryor WA (1986). Oxy-radicals and related species :their formation, lifetime and reactions. Ann Rev Physiol.

[15] Ciencewicki J, Trivedi S, Kleeberger SR (2008). Oxidants and the pathogenesis of lung diseases. J Allergy Clin Immunol.

[16] Church T, Pryor WA (1985). Free-radical chemistry of cigarette smoke and its toxicological implications. Environ Health Perspect.

[17] MacNee W, Wiggs B, Belzberg AS, Hogg JC (1989). The effect of cigarette smoking on neutrophil kinetics in human lungs. N Engl J Med.

[18] Di Stefano A, Maestrelli P, Roggeri A, Turato G, Calabro S, Potena A (1994). Upregulation of adhesion molecules in the bronchial mucosa of subjects with chronic obstructive bronchitis. Am J Respir Crit Care Med.

[19] Schaberg T, Haller H, Rau M, Kaiser D, Fassbender M, Lode H (1992). Superoxide anion release induced by platelet-activating factor is increased in human alveolar macrophages from smokers. Eur Respir J.

[20] MacNee W, Rahman I (2001). Is oxidative stress central to the pathogenesis of chronic obstructive pulmonary disease?. Trends Mol Med.

[21] Rahman I, Adcock IM (2006). Oxidative stress and redox regulation of lung inflammation in COPD. Eur Respir J.

[22] Fischer BM, Pavlisko E, Voynow JA (2011). Pathogenic triad in COPD: oxidative stress, protease-antiprotease imbalance, and inflammation. Int J Chron Obstruct Pulmon Dis.

[23] Hillas G, Nikolakopoulou S, Hussain S, Vassilakopoulos T (2013). Antioxidants and mucolytics in COPD management: when (if ever) and in whom ?. Curr Drug Targets.

[24] Lundborg M, Bouhafs R, Gerde P, Ewing P, Camner P, Dahlen SE (2007). Aggregates of ultrafine particles modulate lipid peroxidation and bacterial killing by alveolar macrophages. Environ Res.

[25] Kienast K, Knorst M, Lubjuhn S, Muller-Quernheim J, Ferlinz R (1994). Nitrogen dioxide-induced reactive oxygen intermediates production by human alveolar macrophages and peripheral blood mononuclear cells. Arch Environ Health.

[26] Droge W (2002). Free radicals in the physiological control of cell function. Physiol Rev.

[27] Lakshminarayan V, Drab-Weiss EA, Roebuck KA (1998). H_2_O_2_ and tumor necrosis factor alpha induce differential binding of the redox-responsive transcription factor AP-1 and NF-kappa B to the interleukin 8 promoter in endothelial and epithelial cells. J Biol Chem.

[28] MacNee W (2000). Oxidants/Antioxidants and COPD. Chest.

[29] Fuke S, Betsuyaku T, Nasuhara Y, Morikawa T, Katoh H, Nishimura M (2004). Chemokines in bronchiolar epithelium in the development of chronic obstructive pulmonary disease. Am J Respir Cell Mol Biol..

[30] Li XY, Donaldson K, Rahman I, MacNee W (1994). An investigation of the role of glutathione in the increased epithelial permeability induced by cigarette smoke *in vivo* and *in vitro*. Am J Respir Crit Care Med.

[31] Morrow JD, Frei B, Longmire AW, Gaziano JM, Lynch SM, Shyr Y (1995). Increase in circulating products of lipid peroxidation (F2-isoprostanes) in smokers. N Engl J Med.

[32] Rahman I, van Schadewijk AA, Crowther AJ, Hiemstra PS, Stolk J, MacNee W (2002). 4-Hydroxy-2-nonenal, a specific lipid peroxidation product, is elevated in lungs of patients with chronic obstructive pulmonary disease. Am J Respir Crit Care Med.

[33] Sadowska AM, van Overveld FJ, Górecka D, Zdral A, Filewska M, Demkow UA (2005). The interrelationship between markers of inflammation and oxidative stress in chronic obstructive pulmonary disease : modulation by inhaled steroids and antioxidant. Respir Med.

[34] Yao H, Rahman I (2011). Current concepts on oxidative/carbonyl stress, inflammation and epigenetics in pathogenesis of chronic obstructive pulmonary disease. Toxicol Appl Pharmacol..

[35] Wozniak A, Gorecki D, Szpinda M, Mila-Kierzenkowska C, Wozniak B (2013). Oxidant-antioxidant balance in the blood of patients with chronic obstructive pulmonary disease after smoking cessation. Oxid Med Cell Longev..

[36] Ahmad A, Shameem M, Husain Q (2013). Altered oxidant-antioxidant levels in the disease prognosis of chronic obstructive pulmonary disease. Int J Tuberc Lung Dis..

[37] Tse HN, Tseng CZS (2014). Update on the pathological processes, molecular biology and clinical utility of N-acetylcysteine in chronic obstructive pulmonary disease. Int J Chron Obstruct Pulmon Dis.

[38] Nicks ME, O’Brien MM, Bowler RP (2011). Plasma antioxidants are associated with impaired lung function and COPD exacerbations in smokers. COPD..

[39] Yao H, Arunachalam G, Hwang JW, Chung S, Sundar IK, Kinnula VL (2010). Extracellular superoxide dismutase protects against pulmonary emphysema by attenuating oxidative fragmentation of ECM. Proc Natl Acad Sci U S A..

[40] Kensler TW, Wakabayashi N, Biswal S (2007). Cell survival responses to environmental stresses via the Keap1-Nrf2-ARE pathway. Annu Rev Pharmacol Toxicol..

[41] Malhotra D, Thimmulappa R, Navas-Acien A, Sandford A, Elliott M, Singh A (2008). Decline in NRF2-regulated antioxidant in chronic obstructive pulmonary disease lungs due to loss of its positive regulator DJ-1. Am J Respir Crit Care Med.

[42] Rangasamy T, Cho CY, Thimmulappa RK, Zhen L, Srisuma SS, Kensler TW (2004). Genetic ablation of Nrf2 enhances susceptibility to cigarette smoke-induced emphysema in mice. J Clin Invest.

[43] Rahman I, Li XY, Donaldson K, Harrison DJ, MacNee W (1995). Glutathione homeostasis in alveolar epithelial cells in vitro and lung in vivo under oxidative stress. Am J Physiol.

[44] Ito K, Ito M, Elliott WM, Cosio B, Caramori G, Kon OM (2005). Decreased histone deacetylase activity in chronic obstructive pulmonary disease. N Engl J Med.

[45] Wada H, Takizawa H (2013). Future treatment for COPD: Targeting oxidative stress and its related signal. Recent Pat Inflamm Allergy Drug Discov.

[46] Drost EM, Skwarski KM, Sauleda J, Soler N, Roca J, Agusti A (2005). Oxidative stress and airway inflammation in severe exacerbations of COPD. Thorax.

[47] Stanojkovic I, Kotur-Stevuljevic J, Milenkovic B, Spasic S, Vujic T, Stefanovic A (2011). Pulmonary function, oxidative stress and inflammatory markers in severe COPD exacerbation. Respir Med.

[48] Britton JR, Pavord ID, Richards KA, Knox AJ, Wisniewski AF, Lewis SA (1995). Dietary antioxidant vitamin intake and lung function in the general population. Am J Respir Crit Care Med..

[49] Grievink L, Smit HA, Ocke MC, van't Veer P, Kromhout D (1998). Dietary intake of antioxidant (pro)-vitamins, respiratory symptoms and pulmonary function: the MORGEN study. Thorax.

[50] Rautalahti M, Virtamo J, Haukka J, Heinonen OP, Sundvall J, Albanes D (1997). The effect of alpha-tocopherol and beta-carotene supplementation on COPD symptoms. Am J Respir Crit Care Med..

[51] Sargeant LA, Jaeckel A, Wareham NJ (2000). Interaction of vitamin C with the relation between smoking and obstructive airways disease in EPIC Norfolk. European Prospective Investigation into Cancer and Nutrition. Eur Respir J.

[52] Tabak C, Arts IC, Smit HA, Heederik D, Kromhout D (2001). Chronic obstructive pulmonary disease and intake of catechins, flavonols, and flavones: the MORGEN Study. Am J Respir Crit Care Med..

[53] Culpitt SV, Rogers DF, Fenwick PS, Shah P, De Matos C, Russell RE (2003). Inhibition by red wine extract, resveratrol, of cytokine release by alveolar macrophages in COPD. Thorax.

[54] Robb EL, Page MM, Wiens BE, Stuart BA (2008). Molecular mechanisms of oxidative stress resistance induced by resveratrol: specif and progressive induction of MsSOD. Biochem Biophys Res Commun.

[55] Kode A, Rajendrasozhan S, Caito S, Yang SR, Megson IL, Rahman I (2008). Resveratrol induces glutathione synthesis by activation of Nrf2 and protects against cigarette smoke-mediated oxidative stress in human lung epithelial cells. Am J Physiol Lung Cell Mol Physiol.

[56] Sheffner AL (1963). The reduction in vitro in viscosity of mucoprotein solutions by a new mucolytic agent. N acetyl-L-cysteine. Ann NY Acad Sci.

[57] Sadowska AM (2012). N-Acetylcysteine mucolysis in the management of chronic obstructive pulmonary disease. Ther Adv Respir Dis..

[58] Grassier B, Cabanis A, Lebegue S, Brunet C, Dine T, Luyckx M (1994). Decrease of hypochlorous acid and hydroxyl radical generated by stimulated human neutrophils : comparison in vitro of some thiol containing drugs. Methods Find Exp Clin Pharmacol.

[59] Aruoma O, Halliwell B, Hoey M, Butler J (1989). The antioxidant action of N-acetylcysteine : its reaction with hydrogen peroxide, hydroxyl radical, superoxide and hypoclorous acid. Free Radic Biol Med.

[60] Moldeus P, Cotgreave IA, Berggren M (1986). Lung protection by a thiol-containing antioxidant: N-acetylcysteine. Respiration.

[61] Bridgeman MME, Marsden M, MacNee W, Flenley DC, Ryle AP (1991). Cysteine and glutathione concentrations in plasma and bronchoalveolar lavage fluid after treatment with N-acetylcysteine. Thorax.

[62] Martensson J, Jain A, Frayer W, Meister A (1989). Glutathione metabolism in the lung: inhibition of its synthesis leads to lamellar body and mitochondrial defects. Proc Natl Acad Sci USA.

[63] Bridgeman MME, Marsden M, Selby C, Morrison D, MacNee W (1994). Effect of N-acetylcysteine on the concentrations of thiols in plasma, bronchoalveolar lavage fluid, and lung tissue. Thorax.

[64] Simon LM, Suttorp N (1985). Lung cell oxidant injury: decrease in oxidant mediated cytotoxicity by N-acetylcysteine. Eur J Respir Dis.

[65] Wagner PD, Mathieu-Costello O, Bebout DE, Gray AT, Natterson PD, Glennow C (1989). Protection against pulmonary 0_2_ toxicity by N-acetylcysteine. Eur Respir J.

[66] Cotgreave IA (1997). N-acetylcysteine: pharmacological considerations and experimental and clinical applications. Adv Pharmacol.

[67] Dekhuijzen PNR (2004). Antioxidant properties of N-acetylcysteine: their relevance in relation to chronic obstructive pulmonary disease. Eur Respir J.

[68] De Caro L, Ghizzi A, Costa R, Longo A, Ventresca GP, Lodola E (1989). Pharmacokinetics and bioavailability of oral acetylcysteine in healthy volunteers. Arzneimittelforschung.

[69] Sadowska AM, Manuel-y-Kenoy B, De Backer WA (2007). Antioxidant and anti-inflammatory efficacy of NAC in the treatment of COPD : discordant in vitro and in vivo dose-effects: a review. Pulm Pharmacol Ther.

[70] Santangelo F (2003). Intracellular thiol concentration modulating inflammatory response: influence on the regulation of cell functions through cysteine prodrug approach. Curr Med Chem.

[71] Rahman I, MacNee W (2000). Regulation of redox glutathione levels and gene transcription in lung inflammation: therapeutic approaches. Free Radic Biol Med.

[72] Grinberg L, Fibach E, Amer J, Atlas D (2005). N-acetylcysteine amide, a novel cell-permeating thiol, restores cellular glutathione and protects human red blood cells from oxidative stress. Free Radic Biol Med.

[73] Mazor D, Golan E, Philip V, Katz M, Jafe A, Ben Zvi Z (1996). Red blood cell permeability to thiol compounds following oxidative stress. Eur J Haematol.

[74] Tsikas D, Sandmann J, Ikic M, Fauler J, Stichtenoth DO, Frolich JC (1998). Analysis of cysteine and N-acetylcysteine in human plasma by highperformance liquid chromatography at the basal state and after oral administration of N-acetylcysteine. J Chromatogr B: Biomed Sci Appl.

[75] Desaki M, Takizawa H, Kasama T, Kobayashi K, Morita Y, Yamamoto K (2000). Nuclear factor-kb activation in silica-induced interleukin 8 production by human bronchial epithelial cells. Cytokine.

[76] Wuyts WA, Vanaudenaerde BM, Dupont LJ, Demedts MG, Verleden GM (2003). Involvement of p38 MAPK, JNK, p42/p44 ERK and NF-kB in IL-1beta-induced chemokine release in human airway smooth muscle cells. Respir Med.

[77] Reddy NM, Kleeberger SR, Bream JH, Fallon PG, Kensler TW, Yamamoto M (2008). Genetic disruption of the Nrf2 compromises cell-cycle progression by impairing GSH-induced redox signaling. Oncogene.

[78] Boutten A, Goven D, Boczkowski J, Bonay M (2010). Oxidative stress targets in pulmonary emphysema: focus on the Nrf2 pathway. Expert Opin Ther Targets.

[79] Dekhuijzen PNR, van Beurden WJC (2006). The role for N-acetylcysteine in the management of COPD. Int J Chron Obstruct Pulmon Dis.

[80] Linden M, Wieslander E, Eklund A, Larsson K, Brattsand R (1988). Effects of oral N-acetylcysteine on cell content and macrophage function in bronchoalveolar lavage from healthy smokers. Eur Respir J.

[81] Jankowska R, Passowicz Muszynska E, Medrala W, Banas T, Marcinkowska A (1993). The influence of n-acetylcysteine on chemiluminescence of granulocytes in peripheral blood of patients with chronic bronchitis. Pneumonol Alergol Pol.

[82] Novak D, Kasielski M, Antczak A, Pietras T, Bialasiewicz P (1999). Increased content of thiobarbituric and acid-reactive substances and hydrogen peroxide in the expired breath condensate of patients with stable chronic obstructive pulmonary disease : no significant effect of cigarette smoking. Respir Med.

[83] De Benedetto F, Aceto A, Dragani B, Spacone A, Formisano S, Cocco R (2000). Validation of a new technique to assess exhaled hydrogen peroxide: results from normals and COPD patients. Monaldi Arch Chest Dis.

[84] Boman G, Backer U, Larsson S, Melander B, Wahlander L (1983). Oral acetylcysteine reduces exacerbation rate in chronic bronchitis: report of a trial organized by the Swedish Society for Pulmonary diseases. Eur J Respir Dis.

[85] Rasmussen JB, Gleenow C (1988). Reduction in days of illness after long-term treatment with Nacetylcysteine controlled-release tablets in patients with chronic bronchitis. Eur Respir J.

[86] British Thoracic Society Research Committee (1985). Oral N-acetylcysteine and exacerbation rates in patients with chronic bronchitis and severe airways obstruction. Thorax.

[87] Grandjean EM, Berthet P, Ruffmann R, Leuenberger P (2000). Efficacy of oral long-term N-acetylcysteine in chronic bronchopulmonary disease: a meta-analysis of published double-blind, placebo-controlled clinical trials. Clin Ther.

[88] Poole PJ, Black PN (2001). Oral mucolytic drugs for exacerbations of chronic obstructive pulmonary disease: systematic review. Br Med J.

[89] Decramer M, Rutten-van Molken M, Dekhuijzen PN, Troosters T, van Herwaarden C, Pellegrino R (2005). Effects of N-acetylcysteine on outcomes in chronic obstructive pulmonary disease (Bronchitis Randomized on NAC Cost- Utility Study, BRONCUS): A randomised placebo-controlled trial. Lancet.

[90] Sutherland ER, Crapo JD, Bowler RP (2006). N-acetylcysteine and exacerbations of chronic obstructive pulmonary disease. COPD.

[91] Schermer T, Chavannes N, Dekhuijzen R, Wouters E, Muris J, Akkermans R (2009). Fluticasone and N-acetylcysteine in primary care patients with COPD or chronic bronchitis. Respir Med.

[92] Kasielski M, Nowak D (2001). Long-term administration of N-acetylcysteine decreases hydrogen peroxide exhalation in subjects with chronic obstructive pulmonary disease. Respir Med.

[93] Gerrits CMJM, Herings RMC, Leufkens HGM, Lammers J-WG (2003). N-acetylcysteine reduces the risk of re-hospitalization among patients with chronic obstructive pulmonary disease. Eur Respir J.

[94] Zuin R, Palamidese A, Negrin R, Catozzo L, Scarda A, Balbinot M (2005). High-dose N-acetylcysteine in patients with exacerbations of chronic obstructive pulmonary disease. Clin Drug Investig.

[95] De Backer J, Vos W, Van Holsbeke C, Vinchurckar S, Claes R, Parizel PM (2013). Effect of high-dose N-acetylcysteine on airway geometry, inflammation, and oxidative stress in COPD patients. Int J Chron Obstruct Pulmon Dis.

[96] Shen Y, Cai W, Lei S, Zhang Z (2013). Effect of high/low dose N-acetylcysteine in chronic obstructive pulmonary disease: a systematic review and meta-analysis. COPD.

[97] Tse HN, Raiteri L, Wong KY, Yee KS, Ng LY, Wai KY (2013). High-dose N-acetylcysteine in stable COPD. The 1-year, double-blind, randomized placebo-controlled HIACE study. Chest.

[98] McFadden ER, Linden DA (1972). A reduction in maximum midexpiratory flow rate. A spirographic manifestation of small airway disease. Am J Med.

[99] Knudson RJ, Lebowitz MD (1978). Maximal mid-expiratory flow (FEF25–75%): normal limits and assessment of sensitivity. Am Rev Respir Dis.

[100] Flenley DC (1988). Chronic obstructive pulmonary disease. Dis Mon.

[101] Pellegrino R, Viegi G, Brusasco V, Crapo RO, Burgos F, Casaburi R (2005). Interpretative strategies for lung function tests. Eur Respir J.

[102] Burgel PR, Bourdin A, Chanez P, Chabot F, Chaouat A, Chinet T (2011). Update on the roles of distal airways in COPD. Eur Respir Rev..

[103] Burgel PR (2011). The role of small airways in obstructive airway diseases. Eur Respir Rev..

[104] Hansen JE, Sun XG, Wasserman K (2006). Discriminating measures and normal values for expiratory obstruction. Chest.

[105] Goldman MD, Saadeh C, Ross D (2005). Clinical applications of forced oscillation to assess peripheral airway function. Respir Physiol Neurobiol.

[106] Kolsum U, Borrill Z, Roy K, Starkey C, Vestbo J, Houghton C (2009). Impulse oscillometry in COPD : identification of measurements related to airway obstruction, airway conductance and lung volumes. Respir Med.

[107] Johnson MK, Birch M, Carter R, Kinsella J, Stevenson RD (2005). Use of reactance to estimate transpulmonary resistance. Eur Respir J.

[108] Stav D, Raz M (2009). Effect of N-acetylcysteine on air trapping in COPD: a randomized placebo-controlled study. Chest..

[109] Suer E, Sayrac S, Sarinay E, Ozturk HE, Turkoz M, Ichinose A (2008). Variation in the attachment of Streptococcus pneumoniae to human pharyngeal epithelial cells after treatment with S carboxymethylcysteine. J Infect Chemother..

[110] Corn SD, Barstow TJ. Effects of oral N-acetylcysteine on fatigue, critical power, and W’ in exercising humans. Respir Physiol Neurobiol. 2011;178:261–8. 10.1016/j.resp.2011.06.020 21740986

[111] Santus P, Corsico A, Solidoro P, Braido F, Di Marco F, Scichilone N (2014). Oxidative stress and respiratory system: clinical reappraisal of N-acetylcysteine. COPD.

[112] Tse HN, Raiteri L, Wong KY, Yee KS, Ng LY, Yee KS (2014). Benefits of high-dose N-acetylcysteine to exacerbation-prone patients with COPD. Chest.

[113] Hurst JR, Vestbo J, Anzueto A, Locantore N, Müllerova H, Tal-Singer R (2010). Evaluation of COPD Longitudinally to Identify Predictive Surrogate Endpoints (ECLIPSE) Investigators. Susceptibility to exacerbation in chronic obstructive pulmonary disease. N Engl J Med.

[114] Wedzicha JA, Brill SE, Allinson JP, Donaldson GC (2013). Mechanisms and impact of the frequent exacerbator phenotype in chronic obstructive pulmonary disease. BMC Med..

[115] Calverley PM, Anderson JA, Celli B, Ferguson GT, Jenkins C, Jones PW (2007). TORCH Investigators. Salmeterol and fluticasone propionate and survival in chronic obstructive pulmonary disease. N Engl J Med.

[116] Tashkin DP, Celli B, Senn S, Burkhart D, Kesten S, Menjoge S (2008). UPLIFT Study Investigators. A 4-year trial of tiotropium in chronic obstructive pulmonary disease. N Engl J Med.

[117] Vogelmeier C, Hederer B, Glaab T, Schmidt H, Rutten-van Mölken MP, Beeh KM (2011). Tiotropium versus salmeterol for the prevention of exacerbations of COPD. N Engl J Med.

[118] Wedzicha JA, Rabe KF, Martinez FJ, Bredenbröker D, Brose M, Goehring UM (2013). Efficacy of roflumilast in the COPD frequent exacerbator phenotype. Chest..

[119] Zheng JP, Kang J, Huang SG, Chen P, Yao WZ, Yang L (2008). Effect of carbocisteine on acute exacerbation of chronic obstructive pulmonary disease (PEACE Study): a randomized placebo-controlled study. Lancet..

[120] Zheng J-P, Wen F-Q, Bai C-X, Wan H-Y, Kang J, Chen P (2014). Twice daily N-acetylcysteine 600 mg for exacerbations of chronic obstructive pulmonary disease (PANTHEON): a randomised, double-blind placebo-controlled trial. Lancet Respir Med.

[121] Anderson D, MacNee W (2009). Targeted treatment in COPD: a multi-system approach for a multi-system disease. Int J Chron Obstruct Pulmon Dis.

[122] Turner RD, Bothamley GH (2014). N-acetylcysteine for COPD : the evidence remains inconclusive. Lancet Respir Med.

[123] Cazzola M, MacNee W, Martinez FJ, Rabe K, Franciosi LG, Barnes PJ (2008). Outcomes for COPD pharmacological trials: from lung function to biomarkers. Eur Respir J.

[124] Cazzola M, Matera MG (2014). N-acetylcysteine in COPD may be beneficial, but for whom ?. Lancet Respir Med.

[125] Criner GJ, Bourbeau J, Diekemper RL, Ouellette DR, Goodridge D, Hernandez P (2015). Prevention of acute exacerbations of COPD. American College of Chest Physicians and Canadian Thoracic Society Guideline. Chest.

[126] Hogg JC, Chu F, Utokaparch S, Woods R, Elliott WM, Buzatu L (2004). The Nature of Small Airway Obstruction in Chronic Obstructive Pulmonary Disease. N Engl J Med.

[127] Sinojia R, Shaikh M, Kodgule R, Bhosale S, Madas S, Vaidya A (2014). Priming of beta-2 agonist and antimuscarinic induced physiological responses induced by 1200 mg/day NAC in moderate to severe COPD patients: a pilot study. Respir Physiol Neurobiol.

